# Early administration of parenteral estrogen suppresses the deleterious local and systemic inflammatory response in severe burns

**DOI:** 10.1186/cc11072

**Published:** 2012-03-20

**Authors:** JG Wigginton, PE Pepe, JW Simpkins, JW Gatson, KG Wigginton, KR Kareem, JP Minei, D Maass

**Affiliations:** 1 University of Texas Southwestern Medical Center, Dallas, TX, USA; 2 University of North Texas, Fort Worth, TX, USA

## Introduction

Soon after severe burns, deleterious cytokines are produced and found in the burned skin, including dead tissue in third-degree injuries. This is followed by a systemic surge in these markers and correlated with subsequent multiorgan failure (MOF). In animal models, this response can be somewhat blunted by early debridement, but such early intervention is not usually feasible in most clinical settings. As estrogen is a powerful anti-inflammatory/anti-apoptotic agent, we tested parenteral 17β-estradiol (E_2_) as a feasible early alternative intervention to dampen the proinflammatory response.

## Methods

Male rats (*n *= 168) were assigned randomly to one of three groups: (1) sham (no) burn (*n *= 8); (2) burn given placebo (*n *= 80); and (3) burn given E_2 _(estrogen). Groups 2 and 3 had 40% TBSA third-degree dorsal burns, early fluid resuscitation and 0.5 mg/kg i.p. estrogen (or placebo) 15 minutes post burn. From each group of 80, eight animals were sequentially sacrificed (and burn tissue and blood sampled for IL-6, TNFα, IL-1β) at one of 10 time points as follows: 0.5, 1, 2, 4, 6, 8, 18 and 24 hours and 7 days (7 days only for the eight shams).

## Results

**Figure 1:**
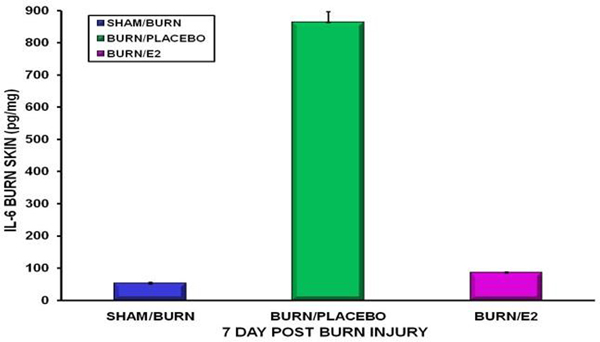
**Burned skin IL-6 levels at day 7**.

In placebos, very high levels of cytokines appeared almost immediately in the echars and circulation, persisting 7 days post burn. In the estrogen group, cytokines, including tissue and circulating IL-6, the greatest predictor of MOF, remained suppressed at all time points, even day 7 (Figure 1).

## Conclusion

Early single-dose parenteral estrogen can dramatically suppress both the local and systemic massive proinflammatory responses in severe burns. Based on these data, estrogen may not only be an inexpensive, simple, adjunctive therapy in burn management, it may obviate the need for many subsequent interventions altogether.
